# Abdominal pain caused by a giant liver hydatid cyst and the management of the residual cavity in a tertiary public hospital in Greece

**DOI:** 10.11604/pamj.2019.34.11.17198

**Published:** 2019-09-04

**Authors:** Athanasios Syllaios, Dimitrios Schizas, Antonios Koutras, Prokopis-Andreas Zotos, Spyridon Davakis, Michail Vailas, Natasha Hasemaki, Evangelia Triantafyllou, Christos Georgiou

**Affiliations:** 1 First Department of Surgery, National and Kapodistrian University of Athens, Laikon General Hospital, Athens, 11527, Greece; 2 Department of Obstetrics and Gynecology, Laikon General Hospital, Athens 11527, Greece; 3 Department of Surgery, University Hospital of Larisa, Larisa 41222, Greece; 4 Department of Surgery, Mpodosakeio Hospital, Ptolemaida 50200, Greece; 5 Department of Surgery, General Hospital of Lamia, Lamia 35100, Greece

**Keywords:** Giant hydatid cyst, liver, residual cavity, echinococcosis, omentoplasty, capitonnage, external tube drainage

## Abstract

Diagnosis and treatment of liver hydatid cysts may be challenging. Many surgical techniques have been proposed for the treatment of liver hydatid cysts, but the problem of the residual cavity still remains controversial and challenging, especially in giant liver hydatid cysts which are rare entities that have not been widely described in the literature so far. Capitonnage, external tube drainage and omentoplasty are the most commonly used techniques. Herein, we report the case of a 70-year-old man with a mild upper quadrant pain that proved to have a giant liver hydatid cyst, 21*14 cm^2^, occupying the entire right lobe of the liver. We describe a successful surgical approach with cyst unroofing and careful evacuation of the multiple daughter cysts by aspiration, and the effective management of the residual cavity by the combination of all three aforementioned techniques.

## Introduction

Cystic echinococcosis is a zoonotic parasitic disease that is caused by Echinococcus granulosus [1]. Liver hydatid cysts (the most common site of occurrence in humans, 50% - 93%) that are not treated can have serious complications: fistulas with adjacent organs or the biliary system, rupture into the peritoneal cavity, or rarely death [2, 3]. Clinical diagnosis may be challenging as mild upper right quadrant pain may be the only symptom. Many surgical techniques have been proposed for the treatment of liver hydatid cysts, but the management of the residual cavity still remains challenging and controversial. Only a few cases of giant liver hydatid cysts have been reported so there are no specific treatment guidelines. We report the case of a 70-year-old man with a giant liver hydatid cyst and a successful surgical approach by cyst unroofing, evacuation of the daughter cysts and management of the residual cavity by the combination of capitonnage, external tube drainage and omentoplasty.

## Patient and observation

**Figure 1:**
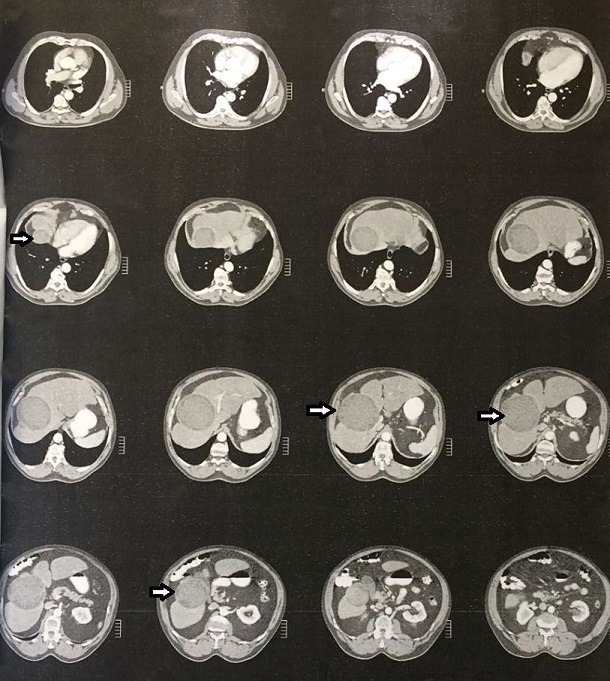
Abdominal CT illustrating the giant liver hydatid cyst (white arrows) occupying the entire right lobe of the liver

A 70-year-old man was referred to the surgical outpatient department because of mild right upper quadrant pain and a sensation of abdominal fullness for about a month. His medical history was unremarkable. The physical examination revealed an enlarged liver with a palpable mass. The plain abdominal X-ray was normal, but the abdominal ultrasonography (US) revealed a large solitary cyst at the right lobe of the liver with multiple cysts inside the primary cyst. The features suggested hydatid aetiology. The diagnosis was confirmed with serological tests, where positive IgG and IgM antibodies were found. An abdominal computed tomography (CT) scan showed a giant, 21*14 cm^2^, marginated hydatid cyst, occupying the entire right lobe of the liver with multiple daughter cysts inside the primary cyst (Figure 1). Communication with major intrahepatic vascular and biliary structures was not found.

We decided not to perform a radical surgical procedure such as cystectomy or major hepatic resection, because of the large size of the hydatid cyst and in order to preserve the liver parenchyma. A small Kocher incision was conducted and the operative field was protected from any spillage to reduce the risk of intraperitoneal soiling and contamination by using gauze packs soaked in 20% hypertonic saline. Cyst unroofing and careful evacuation of the multiple daughter cysts by aspiration with a closed system suction device was performed. 10% povidine iodine was also injected in the giant hydatid cyst for 10 minutes. Biliary orifices were sutured with 3/0 PGA (polyglycolic acid) sutures. Because of the large size of the giant hydatid’s cyst residual cavity capitonnage was not enough and a combination of capitonnage, omentoplasty and external tube drainage was performed. First, the anterior wall of the cyst was sutured in the middle of the posterior wall with absorbable sutures. The rest of the cavity was managed with the drainage tube and a viable pedicle flap of omentum, which was sutured on the cyst’s anterior and posterior wall with absorbable sutures.

The postoperative recovery was uneventful and the patient’s tube drainage was removed on postoperative Day 5. There was no bile fistula. Albendazole 10 mg/kg for 3 cycles was initiated to avoid recurrence. The patient was discharged on postoperative Day 5. After 1 year of follow-up the patient is well and the giant’s hydatid cyst residual cavity has obliterated.

## Discussion

Treatment of cystic echinococcosis can be divided into interventional and non-interventional methods. The first consist of surgery and percutaneous procedures (PAIR), while the second is based on administration of antiparasitic drugs such as albendazole, mebendazole and praziquantel that are used for inoperable patients or for decreasing the recurrence after surgery [1]. Surgery is the treatment of choice for symptomatic and complicated cysts (rupture, compression of vital organs and vessels, secondary bacterial infection, hemorrhage, cysto-biliary fistulas) [2]. Surgical procedures are divided into conservative techniques and radical surgical procedures. Conservative techniques such as marsupialization (the most commonly used) can be used for uncomplicated hydatid cysts, however, the risk of postoperative (bile leak from cyst-biliary communication, bilomas and bile peritonitis) complications is high reaching 4% - 28% On the other hand, radical surgical procedures include cystectomy, pricystectomy, lobectomy and hepatectomy. They have a lower erate of complications and recurrences, but, intraoperative risks are very high. Cystectomy is simple, but, the management of the residual cavity is challenging. Pericystectomy is more difficult and is associated with considerable blood loss. High rates of morbidity and mortality can be found in hepatic resections [4]. Laparoscopic approach is feasible in hepatic hydatid surgery [5]. Advantages of this approach are lower morbidity, shorter hospital stay, faster surgery and better detection of biliary fistula, while disadvantages are higher risks of peritoneal spillage due to high intraabdominal pressures and allergic reactions [4]. In our patient we decided to perform a more conservative technique because of the large size of his hydatid cyst, his old age and in order to preserve the liver parenchyma minimizing the intraoperative and postoperative risks following a major surgical procedure.

The management of the hydatid cyst’s residual cavity still remains a challenging problem, especially in giant hydatid cysts. Many ways of managing the residual cavity have been proposed depending on the size and location of the cyst, the presence or absence of complications and the surgeon’s experience: omentoplasty, external drainage, capitonnage and different combinations of them [6, 7]. Omentoplasty has the lowest surgical site infection (SSI) rate among all other surgical techniques even in combination with the other surgical techniques. Bile fistulas, recurrence rate and overall morbidity is also lower with omentoplasty. As compared to external tube drainage, omentoplasty has also lesser chances of cavity infection, bile leakage and decreased postoperative pain [8]. On the other hand, Manterola et al. mentioned that managing the residual cavity with cappitonage is associated with a lower postoperative morbidity risk than omentoplasty [9]. Omentoplasty and capitonnage have been proposed to be superior to external drainage [6]. As there is no specific management of choice in such situations, we decided to use all three techniques due to the large size of the residual cavity of the hydatid cyst gaining this way total control of the cavity, minimizing the cavity´s gap and the chances of abscess formation and bile leakage.

## Conclusion

In conclusion, setting the diagnosis of liver hydatid cysts may be challenging as mild pain may only exist. In our patient the size and the location of the giant hydatid cyst led us to decide using a combination of capitonnage, omentoplasty and external tube drainage for managing the residual cavity. There are only a few reported cases of giant liver hydatid cysts and there is not an established consensus in managing the residual cavity in those situations, but using the combination of omentoplasty, capitonnage and external tube drainage seems to be a safe and effective way in selected patients.

## Competing interests

The authors declare no competing interests.
